# Spinal Cord Injury Epidemiology and Causes: A Worldwide Analysis with 2050 Projections

**DOI:** 10.3390/healthcare13202552

**Published:** 2025-10-10

**Authors:** Minyoung Kim, Woonyoung Jeong, Suho Jang, Jin Hoon Park, Youngoh Bae, Seung Won Lee

**Affiliations:** 1 Department of MetaBioBealth, School of Medicine, Sungkyunkwan University, Suwon 16419, Republic of Korea; dbssus123@g.skku.edu (M.K.); hbm06014@skku.edu (W.J.); 2 Department of Precision Medicine, School of Medicine, Sungkyunkwan University, Suwon 16419, Republic of Korea; sh.jang.medi@skku.edu; 3 Department of Neurological Surgery, Asan Medical Center, College of Medicine, University of Ulsan, Seoul 05505, Republic of Korea; spinejhpark@naver.com; 4 Department of Neurosurgery, Korean Armed Forces Capital Hospital, Seongnam 13487, Republic of Korea; 5 Department of Family Medicine, Kangbuk Samsung Hospital, School of Medicine, Sungkyunkwan University, 29 Saemunan-ro, Jongno-gu, Seoul 03181, Republic of Korea

**Keywords:** spinal cord injuries, global burden, GBD, prevalence, YLD, projections

## Abstract

**Background/Objectives**: The global burden of spinal cord injury (SCI) is increasing due to aging populations and persistent regional disparities, highlighting an urgent need for updated epidemiological data. This study quantifies the global, regional, and national burden of SCI from 1990 to 2021 and projects its prevalence to 2050. **Methods**: Using data from the Global Burden of Disease (GBD) 2021 study, we estimated age-, sex-, and location-specific prevalence and years lived with disability (YLDs). Projections were developed using sociodemographic modeling, with analyses including Bayesian meta-regression (DisMod-MR 2.1) and Das Gupta decomposition. **Results**: In 2021, approximately 14.5 million people worldwide were living with SCI, including 7.30 million with neck-level and 7.22 million with below-neck-level injuries. The age-standardized prevalence per 100,000 people was 88 for neck-level SCI and 95 for below-neck-level SCI. Although age-standardized rates declined slightly from 1990 (−0.17% for neck-level and −0.18% for below-neck-level), the absolute burden increased substantially. This increase was particularly prominent in East Asia and low- and middle-income countries. The highest prevalence was observed in men aged 50–64 years. Projections indicate that global SCI cases will exceed 14.5 million by 2050. **Conclusions**: These findings underscore the growing absolute burden of SCI. Targeted prevention strategies, enhanced rehabilitation services, and equitable healthcare access are crucial to mitigate long-term disability and improve the quality of life for affected populations worldwide.

## 1. Introduction

Spinal cord injury (SCI), defined as structural damage to the spinal cord resulting from trauma, leads to profound lifelong consequences including impaired mobility, loss of independence, and social isolation [1,2]. Beyond individual disability, SCI imposes a substantial socioeconomic burden: more than 60% of adult patients remain unemployed worldwide [3], and costs encompass acute care, surgery, hospitalization, rehabilitation, assistive devices, home modifications, and management of complications [4]. In high-income countries, the estimated lifetime cost per patient ranges from USD 1.5 to 3.0 million, with the majority of costs incurred during the first year following injury [5]. When indirect costs, such as caregiver support and lost income, are included, the economic impact is even greater.

Despite its severity, diagnostic and management systems for SCI remain underdeveloped in many regions, and the true prevalence in low- and middle-income countries is likely underestimated [6,7]. With increasing risk factors—including population aging, osteoporosis, and urbanization—the global burden of SCI is projected to grow markedly in the coming decades [8].

Recent Global Burden of Disease (GBD) analyses have quantified SCI prevalence and years lived with disability (YLDs), enabling long-term and cross-regional comparisons [9]. However, previous studies have largely focused on overall incidence and aggregated estimates. Critical gaps remain in understanding lesion-level burden (neck-level versus below-neck-level), demographic drivers of disease burden, and absolute prevalence as an indicator of health system demand.

Therefore, this study aimed to quantify the global prevalence and disability burden of SCI from 1990 to 2021 and project trends through 2050 using the GBD 2021 data. This study advances beyond previous research in four key aspects: (1) conducting lesion-level analyses distinguishing neck-level from below-neck-level injuries; (2) examining SCI etiology by age, sex, and region; (3) applying Das Gupta decomposition to quantify contributions from population growth, aging, and epidemiological changes; and (4) analyzing risk rates to derive actionable public health insights. These findings are expected to inform prevention and treatment strategies across health systems and provide critical evidence for mitigating the rising global burden of SCI.

## 2. Materials and Methods

### 2.1. Study Overview

This study utilized data from GBD 2021 to estimate the global prevalence, mortality, and years lived with disability (YLDs) of spinal cord injury (SCI) by age, sex, year, and location, and to project prevalence through 2050. The analysis adhered to the standardized GBD protocol and followed the Guidelines for Accurate and Transparent Health Estimates Reporting (GATHER) [10].

### 2.2. Data Sources and Collection

Data sources for GBD 2021 were obtained from the Global Health Data Exchange (GHDx). Estimates of SCI prevalence and YLDs for 1990–2021 were derived from population-based studies, global and national health surveys, and contributions from collaborating researchers. The dataset encompassed all age groups, sexes, and geographic locations, incorporating records from major SCI causes including transport injuries, falls, and violence. Each data source was assigned a unique identifier and cataloged within the GHDx.

### 2.3. Definition of Case and Causes

SCI was defined as structural disruption of the spinal cord arising from traumatic or non-traumatic causes and resulting in physical impairment. ICD-9 and ICD-10 codes were used to define SCI within the GBD framework. The GBD framework distinguishes between causes of injury (initiating external factors such as falls and road traffic collisions) and nature of injury (consequent health outcomes such as SCI). This investigation focused on the nature of injury [11]. Total SCI prevalence estimates were modeled independently in GBD, with cases classified as neck-level or below-neck-level. Consequently, direct summation of lesion-level estimates may result in slight overcounting and requires cautious interpretation.

### 2.4. Modeling and Data Processing

Bayesian meta-regression models were fitted using R version 4.4.1 and brms version 2.22.0 to estimate age-, sex-, location-, and year-specific prevalence and YLDs [12] (Table S1). YLDs were calculated by multiplying prevalence by a single disability weight representing complete motor function loss, which was applied uniformly across both neck-level and below-neck-level SCI. Data lacking sex-specific or granular age information were disaggregated using prior distributions, a female-to-male prevalence ratio of 1.19 (95% uncertainty interval [UI] 1.03–1.40), and GBD 2021 age patterns. Alternative case definitions were harmonized using MR-BRT network meta-analysis. Outliers were excluded when their absolute deviation from the mean exceeded 1.5 times the mean absolute deviation (MAD), defined as MAD = mean(|x − mean|). Additional Bayesian meta-regression using DisMod-MR 2.1 estimated prevalence and YLDs across demographics (Meta-Regression—Bayesian, Regularized, Trimmed). The prevalence under age 5 was assumed to be zero, consistent with prior GBD analyses and supported by the very low number of reported cases in pediatric SCI literature [13]. Uncertainty intervals (UIs) reflect the 2.5th–97.5th percentiles of 1000 posterior draws. Age-standardized prevalence and YLDs were calculated using the GBD world standard population, enabling consistent comparisons across years, sexes, and locations. Estimates are reported for 7 super-regions, 21 regions, and 204 countries. In the GBD framework, mortality is attributed to SCI only when SCI is the direct cause of death. The final model is as follows:

$$Logit\;\left(predicted\;prevalence\right)=\;\beta_{1}\left(SDI\right)+\;\alpha_{l,a,s}$$

where *β*_1_ is the fixed effect of the sociodemographic index (*SDI*), and *α* denotes random intercepts for location (*l*), age group (*a*), and sex (*s*). Projections through 2050 were calibrated using the 2020 GBD values. Das Gupta partitioned the projected increase in cases (2020–2050) into components attributable to population growth, aging, and non-demographic prevalence changes. Model validation used data from 1990 to 2020 to predict trends through 2050.

### 2.5. Risk Estimation

GBD 2021 includes risk factors relevant to SCI—falls, road injuries, other transport injuries, police conflict, and violence. In older adults, even low-energy trauma may elevate the risk of osteoporosis-related fractures. These risks were incorporated into DisMod-MR 2.1 using standardized covariates derived from summary exposure values (SEVs).

### 2.6. Estimation of Future Prevalence

Future prevalence through 2050 was projected by regressing age-, location-, and sex-specific prevalence estimates from 1990 to 2021 against the SDI. These projections were further refined by adjusting the predicted prevalence rates to the forecasted population estimates. To improve projection accuracy, we calibrated future trends using the difference between predicted 2020 values and actual GBD 2021 estimates. Furthermore, Das Gupta decomposition was applied to partition changes in case counts (2020–2050) into three components: population growth, population aging, and epidemiological changes independent of demographic shifts [14].

## 3. Results

This study assessed the global, regional, and national burden of SCI from 1990 to 2021, including age- and sex-specific prevalence rates, major injury-related risk factors, and projections through 2050.

### 3.1. Global Prevalence in 2021

**Figure 1:**
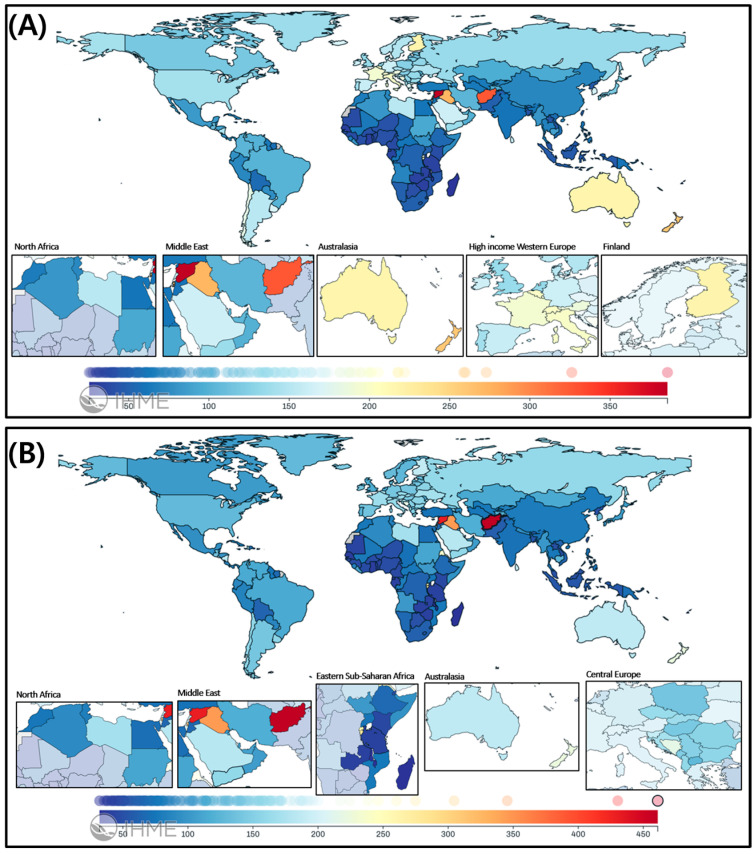
Global distribution of spinal cord injuries in 2021: Geographic patterns and sex disparities. (**A**) Spinal cord lesion at neck level and (**B**) Spinal cord lesion below neck level. Each map displays the age-standardized incidence rate (per 100,000 population) of spinal cord injuries (SCIs) in 2021, stratified by lesion level, across 204 countries and territories.

In 2021, an estimated 7.42 million individuals (95% UI: 6.74–8.35 million) were living with neck-level SCI, representing an increase in absolute counts compared with 1990 (Table 1A, Figure 1A). The age-standardized prevalence reached 88 per 100,000 population (95% UI, 80–100), reflecting a slight decline versus 1990 (relative change: −0.17%, 95% UI, −0.18 to −0.15). The age-standardized YLDs were 35 per 100,000 (95% UI, 25–45), also showing a modest decrease (relative change: −0.21%, 95% UI, −0.21 to −0.19).

For below-neck-level SCI, an estimated 7.98 million individuals (95% UI: 7.15–9.16 million) were affected in 2021 (Table 1B, Figure 1B). The age-standardized prevalence rate was 95 per 100,000 population (95% UI: 85–109), showing a decrease of −0.18% (95% UI: −0.20% to −0.14%) from 1990, while YLDs demonstrated a −0.29% (95% UI: −0.30% to −0.27%) reduction to 20 per 100,000 population (95% UI: 14–28).

### 3.2. Regional Variation in Prevalence

#### 3.2.1. Neck-Level SCI

Australasia had the highest age-standardized prevalence at 225 per 100,000 population (95% UI: 199–253), followed by High-income North America (139 per 100,000), Eastern Europe (136 per 100,000), and Central Europe (134 per 100,000) (Table 1A; Figure 1A). From 1990, Eastern Europe showed a slight relative decline (−0.20%, 95% UI, −0.21 to −0.20), whereas increasing trends were observed in the Caribbean (+0.49%), Oceania (+0.37%), and Western Sub-Saharan Africa (+0.14%).

#### 3.2.2. Below-Neck-Level SCI

Australasia also recorded the highest prevalence at 193 per 100,000 population (95% UI, 172–216), followed by Eastern Europe (160), Central Europe (153), and Southern Latin America (147) (Table 1B; Figure 1B). Increasing trends were observed in the Caribbean (+0.49%), Oceania (+0.33%), and Central Sub-Saharan Africa (+0.24%).

#### 3.2.3. Country-Level Prevalence and YLDs for SCI

At the national level, Afghanistan exhibited the highest age-standardized prevalence in 2021—41.5 per 100,000 for total SCI (20.2 for neck-level and 21.3 for below-neck-level) (Figure 1). Yemen ranked second, with 22.5, 11.1, and 11.4 per 100,000, respectively. Regionally, the burden was greatest in North Africa, the Middle East, Eastern Europe, and Central Asia, as shown in the insets of Figure 1.

#### 3.2.4. Age- and Sex-Specific Prevalence in 2021

**Figure 2:**
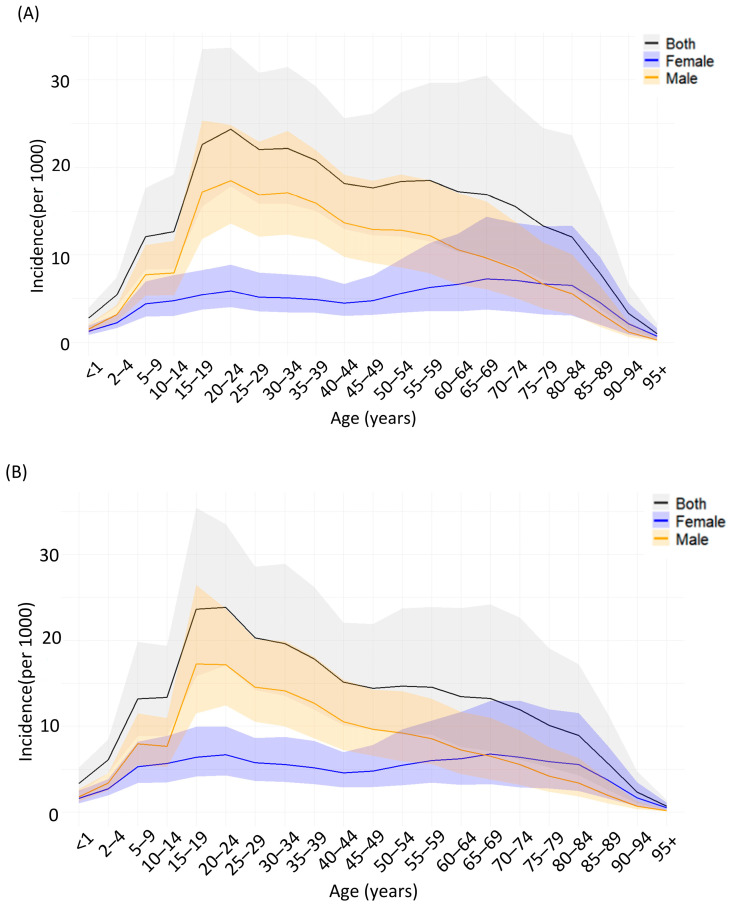
Age- and sex-specific incidence of spinal cord injuries globally in 2021. (**A**) Incidence of neck-level spinal cord injuries per 100,000 population by age group and sex. (**B**) Incidence of below-neck-level spinal cord injuries per 100,000 population by age group and sex. Solid lines represent point estimates for both sexes combined (black), females (blue), and males (orange). Shaded areas indicate 95% uncertainty intervals.

Figure 2 show pronounced sex disparities in SCI prevalence in 2021. Males accounted for 64.2% of all prevalent SCI cases globally, with male predominance particularly marked in the Middle East. The highest male shares were observed in Qatar (89.9%), the United Arab Emirates (89.4%), and Saudi Arabia (83.5%). This pattern was consistent for both neck-level and below-neck-level lesions.

**Figure 3:**
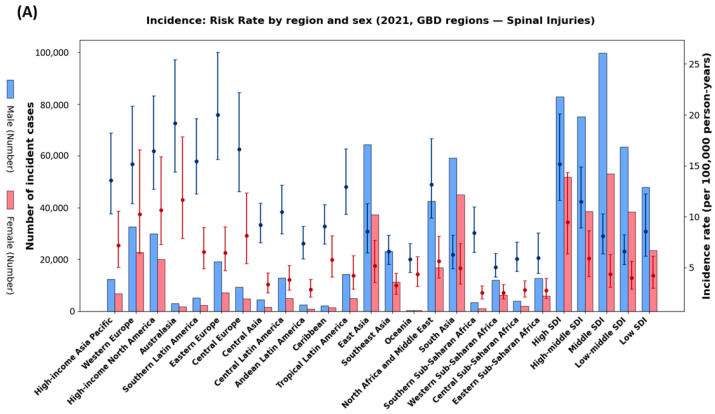

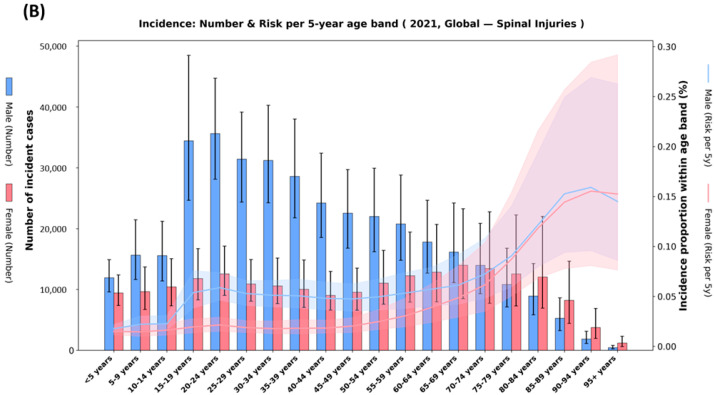
Regional and age-specific incidence of spinal cord injuries in 2021. (**A**) Incidence rates (per 100,000 person-years) and absolute incident cases across GBD regions by sex. Points with error bars represent incidence rates with 95% uncertainty intervals; bars indicate the number of incident cases. (**B**) Global incidence by 5-year age groups showing absolute incident cases (bars) and age-specific incidence proportions (lines with shaded 95% uncertainty intervals). Blue represents males and red/pink represents females. Shaded areas indicate 95% uncertainty intervals.

The global incidence of spinal cord injuries in 2021 shows distinct variations according to age and sex (Figure 2). Neck-level injuries peaked in males aged 15–24 years, whereas below-neck-level injuries progressively increased among elderly women, surpassing men after age 60. Incident cases were most frequent among men aged 20–34 years. SCI risk rates rose after age 60 in both sexes, with women reaching their peak after age 80 (Figure 3B). Regional analysis revealed the highest incidence rates in Sub-Saharan Africa and South Asia, whereas high-income regions demonstrated relatively lower incidence rates but substantial absolute case numbers owing to aging populations (Figure 3A).

#### 3.2.5. Projections of SCI by 2050

By 2050, the global number of SCI cases is projected to reach 7.30 million for neck-level (95% UI: 6.74–9.71 million) and 7.22 million for below-neck-level (95% UI: 4.98–7.92 million), totaling approximately 14.5 million cases worldwide. Age-standardized prevalence is expected to remain approximately 0.08% for both lesion levels (Table 2).

#### 3.2.6. Decomposition of Drivers (Das Gupta)

**Figure 4:**
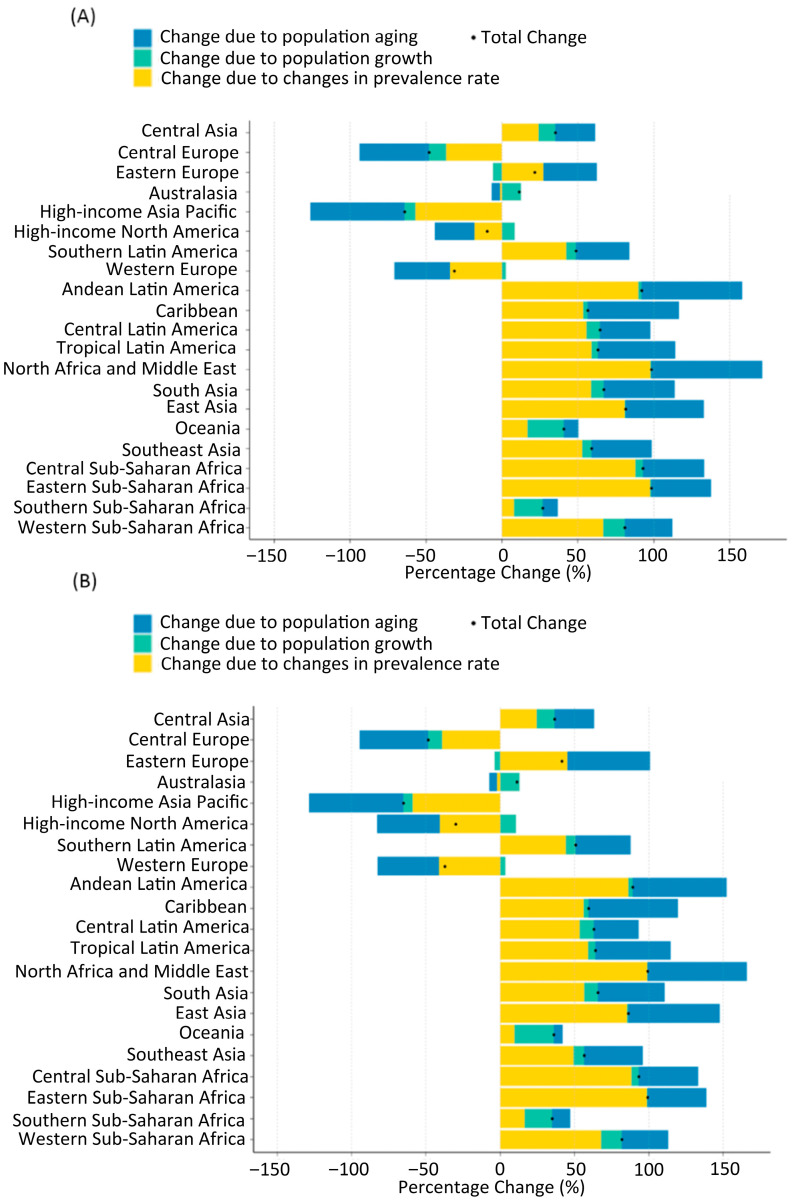
Decomposition of the projected change in the number of prevalent spinal cord lesion cases at the neck level (**A**) and below the neck level (**B**) between 2020 and 2050. Bars represent percentage change attributable to (1) population aging (blue), (2) population growth (green), and (3) change in age-specific prevalence rates (yellow). Positive values indicate an increase, and negative values indicate a decrease in cases attributable to that component. The black dot represents the net percentage change for each region.

Das Gupta decomposition analysis reveals that population aging will be the primary driver of increasing case counts in high-income regions, contributing more than 50% of the total increase (Figure 4). Population growth is projected to be the principal driver in Sub-Saharan Africa, explaining >60% of the projected increase in several regions. Positive contributions from prevalence-rate change are expected in South Asia, Central Asia, and parts of Latin America, reflecting either rising risk or improved case ascertainment. In contrast, negative prevalence changes in Eastern Europe and Australasia partially offset demographic pressures. Overall, the combined effect indicates rapid population aging, as shown in Figure 4.

East Asia is anticipated to bear the largest absolute burden by 2050, with the increase primarily attributable to rapid population aging (blue bars in Figure 4). Additional increases are expected in Central Sub-Saharan Africa, Central Latin America, Tropical Latin America, and Andean Latin America, where population growth (yellow bars) is a major contributing factor. Conversely, Western Europe and High-income North America are projected to experience declines in both age-standardized prevalence and absolute case numbers by 2050. Western Europe is expected to decline to 310,000 neck-level (age-standardized prevalence, 0.07%) and 250,000 below-neck-level (ASR, 0.06%) cases, while Central Europe shows the steepest decline, with only 30,000 neck-level and 40,000 below-neck-level cases projected.

To further address sex-specific differences, we performed decomposition analyses separately for females and males (Figure S1). The overall patterns were consistent with the main findings in Figure 4, but notable sex-specific variations were observed. In East and South Asia, population aging contributed more substantially to YLD increases among females, reflecting longer life expectancy and higher susceptibility to osteoporosis-related SCI. In contrast, in Sub-Saharan Africa, population growth was the principal driver among males, consistent with the younger demographic structure and higher injury exposure. Across high-income regions, both sexes exhibited relatively stable or declining YLD rates, partially offsetting demographic pressures. These findings highlight that, while aggregate trends are broadly similar across sexes, sex-specific analyses provide additional nuance for public health planning.

#### 3.2.7. Model Validation

To evaluate out-of-sample performance, we refitted the model using data from 1990 to 2020 and forecasted age-standardized prevalence for 2021 by region and lesion level. These predictions were compared with observed GBD 2021 values, with absolute errors, relative errors, and 95% prediction-interval coverage summarized in Table S2.

#### 3.2.8. Age-Specific Causes of SCI

**Figure 5:**
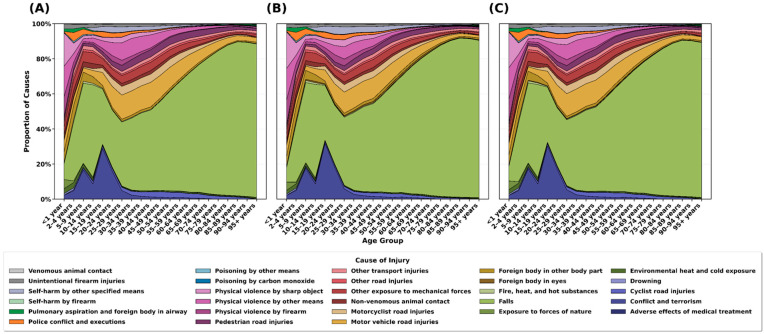
Proportion of causes of spinal cord injuries by age group in 2021. (**A**) Percentage distribution of causes for neck-level spinal cord injuries. (**B**) Percentage distribution of causes for below-neck-level spinal cord injuries. (**C**) Percentage distribution of causes for all spinal injuries combined.

Figure 5A,B illustrate the distribution of SCI causes by age group in 2021, showing clear age-related shifts in etiology across all SCI types.

*Adolescents and young adults (15–35 years).* Road traffic injuries were the predominant cause, accounting for approximately 40–50% of cases. Other transport injuries, interpersonal violence, and self-harm also comprised substantial shares in this age cohort.

*Middle-aged adults (35–65 years).* The proportion of falls increased progressively, emerging as a major cause alongside road-traffic injuries. This period showed transitional characteristics, with a relatively balanced distribution across etiologies.

*Older adults (≥65 years).* Falls became the dominant cause, accounting for ~60–80% of cases—likely reflecting age-related declines in balance, osteoporosis, and muscle weakness.

These age-specific patterns were consistent across lesion levels (neck-level and below-neck-level), although the share of falls was slightly higher in neck-level injuries (Figure 5A vs. Figure 5B).

#### 3.2.9. Sex-Specific Causes of SCI

**Figure 6:**
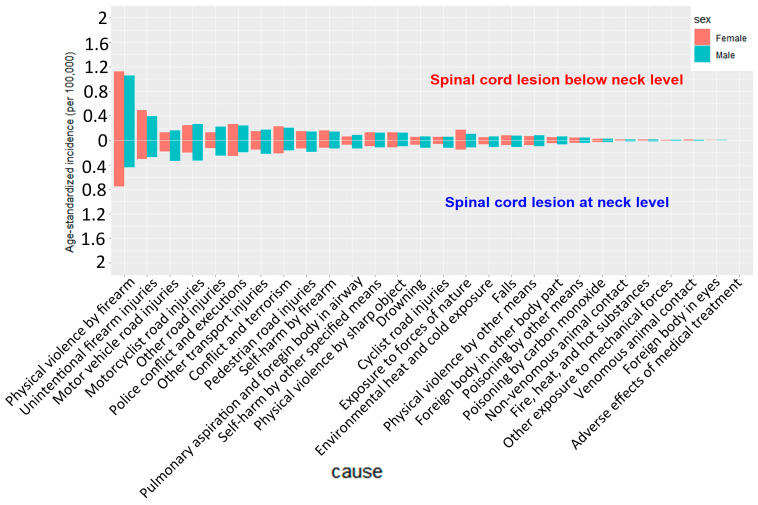
Sex differences in cause-specific age-standardized incidence of spinal cord injuries in 2021.

Pronounced sex disparities in age-standardized incidence by cause were observed in 2021 (Figure 6). Males consistently exhibited higher incidence rates than females across both neck-level and below-neck-level injuries. For below-neck-level injuries (upper panel, Figure 6) “and” For neck-level injuries (lower panel, Figure 6), the most striking sex differences were observed in physical violence by other means and physical violence by firearms, with male incidence rates 3–4 times higher than in females. Motor vehicle and motorcyclist road injuries also showed markedly higher incidence rates in males.

Similar patterns were observed for neck-level injuries (Figure 6, lower panel), although the overall incidence rates were lower than those of below-neck-level injuries. Males demonstrated significantly higher incidence rates of physical violence, traffic accidents, and other transport injuries than females.

Notably, falls exhibited relatively smaller sex differences than other causes, suggesting that falls were more closely associated with age than sex. In contrast, the substantial sex disparities in violence- and traffic-related injuries likely reflect males’ higher risk of exposure and differences in behavioral patterns.

#### 3.2.10. Region-Specific Causes of SCI

**Figure 7:**
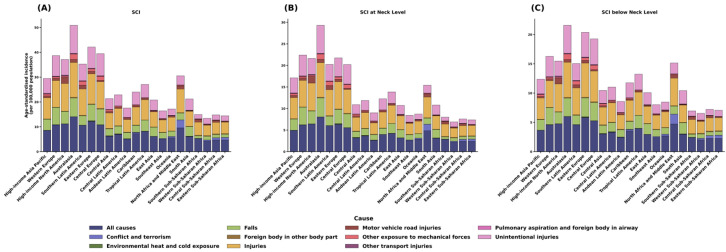
Regional variation in cause-specific age-standardized incidence of spinal cord injuries in 2021. (**A**) Composition of total spinal cord injury incidence by cause across GBD regions. (**B**) Composition of neck-level spinal cord injury incidence by cause. (**C**) Composition of below-neck-level spinal cord injury incidence by cause.

Age-standardized incidence of SCI by cause across 21 GBD regions in 2021 revealed distinct regional variations in etiologic patterns (Figure 7).

#### 3.2.11. High-Income Regions (High-Income Asia Pacific, Western Europe, High-Income North America, Australasia)

Overall incidence ranged from 30 to 50 per 100,000 population, with unintentional injuries and falls (as indicated in the legend; formerly “light purple” and “light green”) as predominant causes. Falls accounted for a substantial share, particularly in Australasia and Western Europe.

#### 3.2.12. Middle-Income Regions (Eastern Europe, Central Europe, Latin America)

Incidence was intermediate (15–30 per 100,000), with a more diverse cause mix. Central and Tropical Latin America showed relatively high proportions of road-traffic (motor-vehicle) and other transport injuries.

#### 3.2.13. Low-Income Regions (Sub-Saharan Africa, South Asia)

Incidence was lower (10–15 per 100,000), potentially reflecting underreporting and limited diagnostic capacity. North Africa and the Middle East displayed a distinctive pattern, with conflict and terrorism comprising a higher share than in other regions.

#### 3.2.14. Comparison by Lesion Level

Similar regional patterns were observed for neck-level and below-neck-level injuries (Figure 7B,C), although neck-level injuries had lower overall incidence. Falls were predominant in East Asia, Central Europe, and High-income North America. In contrast, road traffic injuries comprised the largest proportion in Sub-Saharan Africa and South Asia. Violence-related causes contributed markedly in Latin America and the Caribbean, particularly among younger adults.

#### 3.2.15. Sociodemographic Factors and SCI Prevalence

**Figure 8:**
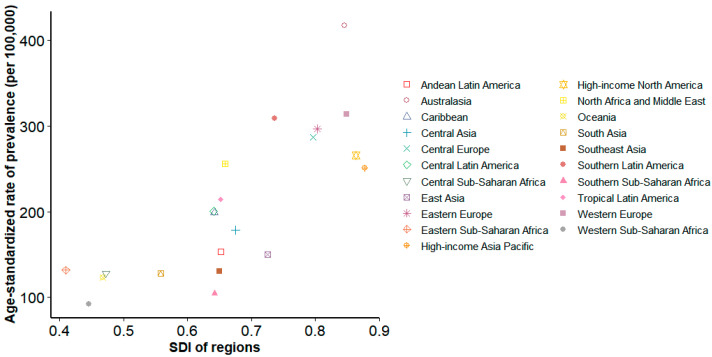
Association between sociodemographic Index (SDI) and age-standardized spinal cord injury prevalence in 2021. Scatter plot showing the relationship between SDI (0–1 scale) and age-standardized injury prevalence per 100,000 population across countries and territories. Each point represents a country/territory, colored by GBD super-region.

The relationship between sociodemographic index (SDI) and age-standardized SCI prevalence in 2021 demonstrated a positive but heterogeneous association across regions (Figure 8). High-income regions with SDI > 0.8 (High-income North America, High-income Asia Pacific, Western Europe) exhibited high prevalence (approximately 200–400 per 100,000 population), likely reflecting older age structures, improved diagnostic ascertainment, and higher post-injury survival. Middle-SDI regions (SDI 0.6–0.7) showed the widest dispersion, with prevalence ranging 100–300 per 100,000 even at comparable SDI levels; Eastern Europe and Latin America fall into this band, where differences in health-system capacity, coding completeness, and trauma exposure may explain variability. Low-SDI regions (SDI 0.4–0.5) generally showed lower prevalence (100–150 per 100,000), although this likely reflects under-ascertainment due to limited diagnostic and reporting infrastructure; Sub-Saharan Africa in particular is prone to underestimation.

## 4. Discussion

### 4.1. Global Prevalence and Burden

Despite slight declines in age-standardized prevalence and YLDs from 1990, the absolute number of people living with SCI has continued to rise due to population growth and aging. Age-standardized prevalence remains high in Eastern Europe, Central and Southern Latin America, and East Asia. High-income countries (HICs) show stabilization or declines in prevalence, while low- and middle-income countries (LMICs) exhibit sustained upward trends.

From 1990 to 2021, crude case counts increased by approximately 0.23% for neck-level and 0.19% for below-neck-level injuries, and YLDs rose by 0.35% and 0.25%, respectively. Consistent with these patterns, global age-standardized prevalence (−0.21%) and age-standardized YLDs (−0.29%) showed modest declines over the study period, even as the absolute number of individuals with SCI-related disability continued to grow. By 2021, the global YLD burden had reached approximately 2.9 million, underscoring the need to strengthen prevention, expand rehabilitation capacity, and improve long-term management.

### 4.2. Social and Economic Impact of SCI

SCI represents both a medical condition and a profound socioeconomic challenge. Patients typically require prolonged rehabilitation and lifelong care, resulting in substantial direct and indirect costs [2]. In high-income countries (HICs), lifetime costs per patient can exceed USD 5 million, with first-year medical expenses alone often surpassing USD 1 million [5]. In low- and middle-income countries (LMICs), inadequate insurance coverage and limited rehabilitation infrastructure shift the financial burden to households. This frequently leads to premature mortality, treatment discontinuation, and “hidden” costs not captured in official statistics [14,15,16]. These disparities exacerbate socioeconomic inequities and underscore the need for equitable resource allocation and policy support across health systems.

### 4.3. Projections of SCI by 2050

Projections indicate a substantial increase in global SCI cases by 2050, driven primarily by population growth and aging. In addition to these demographic drivers, we quantified non-demographic contributions as changes in age-specific prevalence over time, which may reflect shifts in injury incidence, improvements in post-SCI survival, enhanced diagnostic capacity, and better case ascertainment. East Asia is expected to bear the largest burden, as its rapid transition to a super-aged society is likely to increase fall- and osteoporosis-related SCIs. Sub-Saharan Africa and Latin America are also projected to experience rising SCI prevalence, fueled by population expansion, road-traffic injuries, and occupational accidents. In contrast, both prevalence and case counts are expected to decline in Western Europe and North America, consistent with mature prevention policies and stronger healthcare infrastructures. These findings underscore the need for region-specific strategies. In East Asia, priorities include fall-prevention programs, osteoporosis screening integration into primary care, and community-based rehabilitation expansion. In Sub-Saharan Africa and Latin America, essential interventions include strengthening road-safety initiatives (e.g., speed control, helmet laws), building trauma registries, and improving first-responder training. High-income countries, while maintaining current prevention gains, may serve as models for phased implementation in lower-resource settings.

China is projected to face particularly rapid growth in SCI burden due to its unprecedented pace of population aging. To address this, the government has introduced several policy frameworks. The “Healthy China 2030” blueprint identifies fall prevention, osteoporosis management, and expansion of rehabilitation services as core priorities [17]. Integrated health and long-term-care policies aim to expand combined medical–nursing facilities and provide sustained care for chronic conditions such as SCI. In addition, community-based rehabilitation networks are being strengthened to ensure that patients with SCI and other neuromusculoskeletal disorders can access continuous care in secondary hospitals and rehabilitation centers. These initiatives may provide important precedents for East Asia and offer policy lessons for other regions undergoing similar demographic transitions.

### 4.4. Disparities in the Burden of SCI Between HICs and LMICs

Marked disparities persist between high-income countries (HICs) and low- and middle-income countries (LMICs) [18]. According to GBD 2021 estimates, the highest age-standardized prevalence and YLDs were observed in Western and Eastern Europe, high-income North America, and parts of Latin America. These high rates likely reflect population aging and rising degenerative spine disease, as well as greater diagnostic capacity and more extensive imaging use, resulting in improved case ascertainment. However, because our study did not directly analyze these mechanisms, this interpretation should be treated with caution.

By contrast, the SCI burden in LMICs may be substantially underestimated owing to limited healthcare infrastructure and diagnostic capacity [19]. Younger age structures and underdeveloped systems for detecting non-traumatic spinal disorders further exacerbate underestimation. Because population-based SCI registries are scarce, the magnitude of underreporting cannot be precisely quantified; our estimates likely represent a lower bound of the true burden, underscoring the need for improved surveillance and capture–recapture studies.

Although trauma-related causes—falls, road-traffic and occupational injuries—are common, many patients in LMICs have restricted access to acute and rehabilitative care, often resulting in severe disability or premature death [20]. The scarcity of rehabilitation services, assistive devices, and long-term care amplifies the burden beyond what is captured in official statistics [21]. Notably, Afghanistan exhibited the highest age-standardized prevalence in 2021, a finding that may reflect the combined effects of prolonged armed conflict, elevated trauma-related injury rates, limited access to acute and rehabilitation services, and preferential reporting of severe or chronic cases—potentially leading to overestimation in population-based data.

Looking ahead to 2050, LMICs are projected to experience significant increases in SCI prevalence, driven by aging, urbanization, and lifestyle changes, with particularly sharp increases expected in East Asia, Latin America, and Sub-Saharan Africa. This highlights the urgent need for investment in prevention, diagnostic capacity, and rehabilitation infrastructure. Although regional policy frameworks exist, implementation remains limited. The WHO Emergency Care System Framework (2019) outlines strategies to strengthen trauma and emergency care in Sub-Saharan Africa, directly relevant to SCI management [22]. Similarly, the PAHO Road Safety Report (2019) provides targeted interventions to reduce road traffic injuries in Latin America, addressing a leading cause of regional SCI [23]. Expanding and integrating these frameworks into national health systems is essential to mitigate the projected rise in SCI burden.

To further clarify the relationship between sociodemographic development and SCI prevalence, we quantified the correlation between SDI changes and SCI prevalence across 21 GBD regions (Table S3). Most regions demonstrated strong, significant correlations (e.g., Oceania: R^2^ = 0.932, *p* < 0.001; South Asia: R^2^ = 0.829, *p* < 0.001), whereas others—such as Central Sub-Saharan Africa and Central Latin America—showed weak or non-significant associations. These findings underscore the heterogeneity of the SDI–SCI relationship and highlight that socioeconomic progress alone does not uniformly translate into improved SCI outcomes.

### 4.5. Age-Specific Burden of SCI

As demonstrated in this study, the leading causes of SCI vary markedly with age. Among younger adults (15–35 years), nearly half of SCIs are attributable to road-traffic injuries, reflecting greater exposure to automobile/motorcycle driving, outdoor activities, and high-risk sports [7]. A substantial share in this age group is also due to interpersonal violence and self-harm, suggesting contributions from mental-health problems and social instability to SCI incidence in young populations.

In middle-aged adults (35–65 years), falls become increasingly important while road-traffic injuries remain a major contributor, indicating a transitional pattern in etiology. This shift may relate to musculoskeletal degeneration, alcohol use, and balance impairment associated with chronic conditions that commonly emerge in midlife.

In older adults (≥65 years), falls are by far the predominant cause of SCI and are strongly linked to aging, osteoporosis, and muscle weakness [3]. Postmenopausal bone loss renders elderly women particularly susceptible, substantially increasing the risk of vertebral fractures and subsequent SCI [24]. These findings underscore the importance of early diagnosis and treatment of osteoporosis, balance training, and fall-prevention strategies in homes and long-term-care facilities to mitigate the burden of SCI in older populations.

Although neck-level (cervical) and below-neck-level (thoracic/lumbar) SCIs show broadly similar age-related patterns, the proportion of fall-related injuries is higher at the neck level (Figure 6). Cervical SCIs are particularly susceptible to low-energy falls, as ground-level impacts can induce hyperextension or axial loading, resulting in odontoid fractures or central cord syndrome [25]. Pre-existing cervical canal stenosis from spondylosis or OPLL (ossification of the posterior longitudinal ligament) amplifies the risk of cord compression even after minor trauma [26].

By contrast, below-neck-level SCIs more often result from high-energy mechanisms—such as motor vehicle collisions, motorcycle crashes, or axial burst injuries at the thoracolumbar junction—requiring greater force to compromise the thoracic and lumbar spine. Additionally, firearm-related violence contributes disproportionately to below-neck-level injuries, particularly in regions with high interpersonal violence, likely reflecting abdominal and thoracolumbar gunshot wounds that spare the cervical spine [27]. These biomechanical and etiologic differences explain the higher proportion of fall-related injuries at the neck level and the predominance of transport- and violence-related causes below the neck.

### 4.6. Sex Differences in the Burden of SCI

The causes of SCI differ markedly between sexes, with SCIs attributable to physical violence (including firearm-related injuries) and road-traffic injuries (automobile and motorcycle crashes) occurring at 3–4 times the rates in men compared with women, likely reflecting men’s greater exposure to hazardous environments, higher participation in transportation and industrial settings, and increased risk of interpersonal violence [28]. Consistent with this pattern, the World Health Organization has reported that >80% of traumatic SCIs occur in men worldwide [3].

However, an important limitation is that SCIs among women in LMICs may be systematically underdiagnosed and underreported owing to restricted healthcare access, cultural barriers, and socioeconomic constraints. In some regions, women have lower hospital attendance and reduced access to imaging studies, which likely results in an underestimation of their true burden [29]. Thus, the observed male predominance in traumatic SCI may not solely reflect biological or behavioral differences but may also be partially attributable to diagnostic and reporting biases.

In contrast, SCIs caused by falls demonstrated relatively small sex differences. Falls are predominantly associated with aging, osteoporosis, and impaired balance [24], suggesting that physiological vulnerability related to aging rather than sex is a stronger determinant.

In LMICs, SCIs among women may be systematically underdiagnosed and underreported due to barriers to healthcare access, cultural restrictions, and socioeconomic disadvantages [30]. In some regions, limited access to hospitals and delayed treatment hinder accurate case identification, which suggests that the true burden on women may not be fully captured in official statistics. This under-ascertainment may exaggerate the observed male predominance, as a substantial proportion of female cases may go undetected or unrecorded. Cultural norms that restrict women’s mobility, reluctance to seek care for injuries, and the limited availability of female-oriented rehabilitation services may further compound this bias. Consequently, the disproportionately higher rates of traumatic SCI observed in men may not solely reflect differences in risk exposure, but may also be partially influenced by diagnostic and reporting biases [3]. These findings highlight the need for targeted efforts to improve case detection and reporting for women in LMICs, to ensure more accurate sex-specific estimates.

These sex-specific disparities have significant public health implications. For men, preventive measures should emphasize road traffic safety (e.g., motorcycle helmet use and stricter road safety regulations), violence prevention, and occupational safety programs. For women, especially in LMICs, priorities should extend beyond fall and osteoporosis management to include interventions that address barriers to healthcare access, cultural constraints, and socioeconomic inequities. Therefore, SCI prevention and management strategies should adopt tailored approaches that account for sex-, age-, region-, and culture-specific risk factors.

### 4.7. Regional Etiologic Patterns of SCI

In addition to demographic and healthcare factors, region-specific environmental and occupational exposures critically shape SCI etiology. In North Africa and the Middle East, armed conflict and blast-related injuries remain significant causes of SCI, often resulting in severe penetrating lesions [31]. In South Asia and Sub-Saharan Africa, occupational exposures contribute substantially to the SCI burden, particularly falls from height in construction, agriculture, and mining sectors [32]. Conversely, in high-income regions such as North America and Western Europe, a notable proportion of SCIs among young adults are attributable to sports and recreational activities (e.g., diving, rugby, American football), underscoring the need for injury prevention programs targeting these populations [33].

### 4.8. Strengths

- This study provides systematic global, regional, and national estimates of SCI prevalence and YLDs using GBD 2021 data, with projections through 2050.
- Application of Bayesian meta-regression (DisMod-MR 2.1) enabled generation of age-, sex-, and location-specific estimates with high reliability.

### 4.9. Limitations

Despite these strengths, several limitations merit consideration.

- Data and measurement: In many low-income settings, SCI data remain sparse or underreported, potentially reducing estimate accuracy. Cross-national coding differences may also introduce over- or underestimation.
- Diagnostic capacity bias: We could not quantify the correlation between prevalence and diagnostic capacity (e.g., MRI availability, access to care); thus, higher prevalence in HICs may reflect true burden and/or better ascertainment, and should be interpreted cautiously.
- Modeling assumptions: Projections are trend-based and assume stable exposures and policies; we did not model counterfactual scenarios (e.g., improved road safety, fall-prevention scale-up, rehabilitation expansion).
- Sensitivity analyses: Scenario-based sensitivity analyses (e.g., road-safety improvements, aging-policy changes) were not performed.
- Regional extrapolation risk: For regions with volatile recent trends, extrapolation can be implausible. For example, the below-neck-level projection for East Asia over-extrapolated a post-2007 surge and implausibly exceeded the global total by 2050; such outputs require cautious interpretation and potential model constraints.
- Disability weight simplification: Applying a single disability weight to both neck- and below-neck-level injuries may underestimate the higher functional/QoL impact of cervical injuries.
- Validation limits: Systematic per-country validation remains limited due to scarce national registries, although spot checks using extreme-burden countries (Table S4) showed consistency.

## 5. Conclusions

Using GBD 2021 data, this study comprehensively quantified the global prevalence and disability burden of SCI from 1990 to 2021 and projected future trends through 2050. The rising absolute number of cases, particularly in aging populations and LMICs, underscores the urgent need for fall prevention, road safety measures, and expanded rehabilitation services. These findings provide essential evidence to guide targeted prevention, and equitable access to long-term care worldwide.

## Figures and Tables

**Table 1:** Projected age-standardized prevalence and case numbers of spinal cord injuries by region for 2030, 2040, and 2050. (**A**) Neck-level spinal cord injuries. (**B**) Below-neck-level spinal cord injuries.

**(A) Neck-Level Spinal Cord Injuries**						
	**Number of Prevalent Cases**	**Age-Standardized** **Prevalence Rate per** **100,000**	**Percentage Change** **in Age-Standardized** **Prevalence Rate from** **1990 to 2021**	**Number of YLDs**	**Age-Standardized** **Rate of YLDs per** **100,000**	**Percentage Change in** **Age-Standardized Rate** **of YLDs per 100,000** **from 1990 to 2021**
Global	7,423,601 (6,744,305 to 8,352,639)	88 (80 to 100)	−0.17% (−0.18 to −0.15)	2,905,920 (2,070,218 to 3,764,134)	35 (25 to 45)	−0.21% (−0.21 to −0.19)
Central Europe, Eastern Europe, and Central Asia	623,910 (571,153 to 679,453)	125 (114 to 137)	−0.2% (−0.21 to −0.19)	237,632 (169,121 to 303,721)	48 (34 to 62)	−0.24% (−0.26 to −0.23)
Central Asia	80,405 (72,475 to 91,342)	82 (74 to 93)	−0.18% (−0.19 to −0.15)	33,062 (23,144 to 43,389)	34 (23 to 44)	−0.21% (−0.24 to −0.18)
Central Europe	195,626 (178,384 to 215,033)	134 (122 to 148)	−0.12% (−0.13 to −0.12)	72,954 (51,704 to 94,120)	51 (36 to 66)	−0.18% (−0.21 to −0.17)
Eastern Europe	347,878 (317,244 to 379,590)	136 (125 to 150)	−0.2% (−0.21 to −0.2)	131,615 (94,674 to 168,599)	52 (37 to 67)	−0.24% (−0.26 to −0.23)
High-income	2,189,184 (2,004,303 to 2,412,320)	154 (142 to 169)	−0.23% (−0.23 to −0.22)	804,438 (581,439 to 1,035,355)	58 (41 to 75)	−0.25% (−0.25 to −0.24)
Australasia	83,449 (74,836 to 93,591)	225 (199 to 253)	−0.15% (−0.17 to −0.13)	30,802 (21,864 to 40,108)	84 (59 to 111)	−0.16% (−0.16 to −0.15)
High-income Asia Pacific	352,220 (323,624 to 386,775)	135 (124 to 149)	−0.31% (−0.31 to −0.3)	130,234 (93,364 to 168,343)	51 (36 to 66)	−0.33% (−0.34 to −0.32)
High-income North America	674,634 (613,670 to 739,189)	139 (128 to 151)	−0.25% (−0.24 to −0.26)	243,021 (174,278 to 309,329)	51 (36 to 65)	−0.27% (−0.26 to −0.28)
Southern Latin America	121,955 (112,339 to 132,701)	162 (149 to 177)	0.05% (0.03 to 0.05)	47,491 (34,450 to 60,752)	63 (46 to 81)	−0.02% (−0.01 to 0)
Western Europe	956,925 (868,113 to 1,066,230)	169 (152 to 190)	−0.21% (−0.22 to −0.2)	352,889 (249,483 to 459,795)	64 (44 to 84)	−0.22% (−0.24 to −0.21)
Latin America and Caribbean	595,836 (545,422 to 656,732)	94 (86 to 104)	−0.14% (−0.13 to −0.16)	240,204 (169,301 to 312,936)	38 (27 to 49)	−0.19% (−0.2 to −0.17)
Andean Latin America	47,241 (42,378 to 54,760)	72 (64 to 83)	−0.02% (0.01 to −0.06)	19,051 (13,590 to 24,756)	29 (20 to 38)	−0.11% (−0.11 to −0.14)
Caribbean	45,799 (36,150 to 64,892)	92 (72 to 133)	0.49% (0.24 to 1)	19,068 (12,561 to 30,171)	39 (25 to 62)	0.48% (0.36 to 0.89)
Central Latin America	248,604 (225,442 to 281,664)	94 (85 to 106)	−0.27% (−0.26 to −0.3)	100,208 (70,683 to 128,309)	38 (27 to 48)	−0.32% (−0.34 to −0.32)
Tropical Latin America	254,191 (229,709 to 281,264)	101 (91 to 112)	−0.07% (−0.08 to −0.06)	101,877 (73,864 to 132,130)	40 (29 to 53)	−0.13% (−0.14 to −0.1)
North Africa and Middle East	733,916 (557,226 to 1,051,272)	118 (90 to 168)	0.03% (0.03 to 0.03)	295,290 (188,889 to 467,159)	47 (30 to 74)	−0.04% (−0.07 to 0.02)
South Asia	1,043,559 (941,263 to 1,178,872)	58 (53 to 65)	0.09% (0.08 to 0.12)	435,161 (316,813 to 560,991)	24 (18 to 31)	0.02% (0.03 to 0.06)
Southeast Asia, east Asia, and Oceania	1,794,921 (1,656,802 to 1,958,579)	68 (63 to 75)	0.01% (0 to 0.01)	698,114 (497,592 to 896,538)	27 (19 to 35)	−0.09% (−0.1 to −0.05)
East Asia	1,345,847 (1,244,874 to 1,458,414)	71 (66 to 77)	0.04% (0.03 to 0.04)	511,349 (366,019 to 662,719)	27 (19 to 35)	−0.08% (−0.1 to −0.05)
Oceania	6850 (6131 to 7947)	56 (51 to 65)	0.37% (0.33 to 0.45)	3007 (2107 to 3887)	24 (17 to 31)	0.34% (0.34 to 0.36)
Southeast Asia	442,224 (384,239 to 537,680)	60 (52 to 72)	−0.07% (−0.06 to −0.15)	183,757 (130,575 to 251,455)	25 (18 to 34)	−0.13% (−0.11 to −0.15)
Sub-Saharan Africa	442,275 (353,948 to 595,288)	49 (40 to 66)	−0.04% (−0.06 to −0.04)	195,082 (134,468 to 286,237)	21 (15 to 32)	−0.08% (−0.08 to −0.03)
Central Sub-Saharan Africa	60,143 (44,116 to 89,445)	56 (41 to 83)	0.21% (0.13 to 0.31)	26,763 (16,756 to 43,290)	24 (16 to 40)	0.16% (0.11 to 0.24)
Eastern Sub-Saharan Africa	186,103 (133,709 to 291,562)	57 (40 to 89)	−0.03% (0.03 to −0.07)	82,551 (53,111 to 131,717)	25 (16 to 40)	−0.08% (−0.05 to −0.11)
Southern Sub-Saharan Africa	37,233 (34,301 to 40,654)	46 (43 to 51)	−0.43% (−0.42 to −0.44)	15,649 (11,349 to 19,449)	19 (14 to 24)	−0.45% (−0.45 to −0.45)
Western Sub-Saharan Africa	158,796 (138,926 to 191,824)	42 (37 to 49)	0.14% (0.09 to 0.23)	70,119 (50,247 to 92,436)	18 (13 to 24)	0.1% (0.09 to 0.16)
**(B) Below-Neck Spinal Cord Injuries**						
	**Number of Prevalent Cases**	**Age-Standardized** **Prevalence Rate per** **100,000**	**Percentage Change** **in Age-Standardized** **Prevalence Rate from** **1990 to 2021**	**Number of YLDs**	**Age-Standardized** **Rate of YLDs per** **100,000**	**Percentage Change in** **Age-Standardized Rate** **of YLDs per 100,000** **from 1990 to 2021**
Global	7,977,081 (7,150,375 to 9,155,049)	95 (85 to 109)	−0.18% (−0.2 to −0.14)	1,660,317 (1,148,393 to 2,345,733)	20 (14 to 28)	−0.29% (−0.3 to −0.27)
Central Europe, Eastern Europe, and Central Asia	742,302 (677,934 to 820,823)	146 (133 to 162)	−0.21% (−0.21 to −0.2)	135,615 (94,895 to 184,783)	27 (19 to 37)	−0.36% (−0.36 to −0.33)
Central Asia	94,450 (84,900 to 106,458)	97 (87 to 109)	−0.18% (−0.2 to −0.17)	21,884 (15,117 to 30,300)	22 (15 to 31)	−0.28% (−0.3 to −0.26)
Central Europe	227,830 (206,852 to 253,503)	153 (138 to 170)	−0.16% (−0.17 to −0.14)	38,451 (26,486 to 53,726)	26 (18 to 36)	−0.37% (−0.38 to −0.34)
Eastern Europe	420,022 (385,322 to 461,941)	160 (147 to 178)	−0.2% (−0.2 to −0.19)	75,280 (52,094 to 103,014)	29 (20 to 40)	−0.35% (−0.36 to −0.33)
High-income	1,921,663 (1,777,023 to 2,084,318)	135 (124 to 147)	−0.25% (−0.25 to −0.25)	315,500 (219,411 to 431,929)	23 (15 to 31)	−0.3% (−0.32 to −0.3)
Australasia	72,033 (64,899 to 80,741)	193 (172 to 216)	−0.19% (−0.2 to −0.18)	11,789 (8044 to 16,299)	32 (22 to 44)	−0.2% (−0.21 to −0.19)
High-income Asia Pacific	301,367 (279,124 to 331,301)	116 (106 to 128)	−0.33% (−0.34 to −0.32)	49,302 (33,909 to 67,461)	19 (13 to 27)	−0.41% (−0.43 to −0.4)
High-income North America	612,529 (564,982 to 664,508)	126 (117 to 137)	−0.27% (−0.27 to −0.28)	98,250 (67,873 to 131,470)	21 (14 to 28)	−0.3% (−0.3 to −0.31)
Southern Latin America	111,011 (102,952 to 120,875)	147 (136 to 160)	0.01% (−0.01 to 0.01)	21,726 (15,258 to 29,465)	29 (20 to 39)	−0.21% (−0.21 to −0.18)
Western Europe	824,722 (753,071 to 911,533)	145 (131 to 162)	−0.24% (−0.24 to −0.22)	134,433 (92,612 to 184,891)	24 (16 to 33)	−0.28% (−0.3 to −0.27)
Latin America and Caribbean	673,948 (612,096 to 745,341)	107 (97 to 118)	−0.17% (−0.16 to −0.18)	152,452 (105,859 to 203,532)	24 (17 to 32)	−0.33% (−0.34 to −0.32)
Andean Latin America	53,578 (47,491 to 62,396)	82 (72 to 95)	−0.06% (−0.04 to −0.11)	11,873 (8221 to 16,577)	18 (12 to 25)	−0.3% (−0.32 to −0.33)
Caribbean	53,007 (40,972 to 77,927)	107 (81 to 159)	0.49% (0.21 to 1.07)	13,568 (8381 to 21,628)	28 (17 to 44)	0.48% (0.29 to 0.84)
Central Latin America	281,254 (250,868 to 322,430)	106 (95 to 122)	−0.28% (−0.27 to −0.31)	62,900 (43,911 to 87,260)	24 (17 to 33)	−0.43% (−0.43 to −0.43)
Tropical Latin America	286,109 (259,071 to 319,707)	113 (102 to 127)	−0.14% (−0.13 to −0.12)	64,110 (44,576 to 84,822)	25 (18 to 34)	−0.3% (−0.32 to −0.29)
North Africa and Middle East	845,673 (617,408 to 1,306,946)	138 (101 to 212)	0.03% (−0.01 to 0.03)	187,822 (114,166 to 337,074)	30 (19 to 54)	−0.2% (−0.24 to −0.14)
South Asia	1,238,974 (1,101,859 to 1,392,457)	70 (62 to 78)	0.03% (0 to 0.05)	320,436 (226,727 to 437,659)	18 (13 to 24)	−0.16% (−0.16 to −0.12)
Southeast Asia, east Asia, and Oceania	2,009,736 (1,826,885 to 2,218,767)	77 (69 to 85)	−0.04% (−0.06 to −0.04)	387,724 (270,461 to 533,675)	15 (10 to 21)	−0.33% (−0.34 to −0.3)
East Asia	1,482,829 (1,367,846 to 1,625,239)	79 (72 to 86)	−0.03% (−0.03 to −0.01)	258,674 (180,186 to 353,506)	14 (10 to 19)	−0.37% (−0.37 to −0.34)
Oceania	7934 (7043 to 9342)	67 (60 to 77)	0.33% (0.29 to 0.41)	2334 (1642 to 3040)	19 (14 to 25)	0.27% (0.28 to 0.25)
Southeast Asia	518,973 (433,170 to 652,801)	70 (59 to 87)	−0.07% (−0.09 to −0.14)	126,715 (85,919 to 186,473)	17 (12 to 25)	−0.24% (−0.23 to −0.26)
Sub-Saharan Africa	544,784 (419,861 to 788,338)	63 (48 to 91)	−0.01% (−0.06 to 0.06)	160,768 (102,917 to 260,953)	18 (12 to 30)	−0.11% (−0.15 to −0.02)
Central Sub-Saharan Africa	75,010 (52,619 to 119,583)	72 (50 to 115)	0.24% (0.08 to 0.43)	22,722 (13,781 to 40,341)	21 (13 to 38)	0.1% (0.01 to 0.24)
Eastern Sub-Saharan Africa	237,422 (161,438 to 408,142)	75 (50 to 132)	0.03% (0.01 to 0.06)	71,663 (423,69 to 131,261)	22 (13 to 42)	−0.09% (−0.12 to −0.07)
Southern Sub-Saharan Africa	45,354 (41,668 to 49,898)	58 (53 to 63)	−0.38% (−0.38 to −0.39)	11,782 (8392 to 15,513)	15 (11 to 20)	−0.44% (−0.43 to −0.44)
Western Sub-Saharan Africa	186,997 (161,440 to 229,724)	50 (44 to 61)	0.11% (0.05 to 0.24)	54,601 (37,545 to 79,349)	14 (10 to 21)	0% (−0.01 to 0.1)

Data in parentheses are 95% uncertainty intervals. Region and super-region numbers do not sum to the global prevalence due to rounding. YLDs = years lived with disability.

**Table 2:** Projected age-standardized prevalence and case numbers of spinal cord injuries by region for 2030, 2040, and 2050. (**A**) Neck-level spinal cord injuries. (**B**) Below-neck-level spinal cord injuries.

**(A) Neck-Level Spinal Cord Injuries**.						
	**Age-Standardized Prevalence**			**Cases (Millions)**		
	**2030**	**2040**	**2050**	**2030**	**2040**	**2050**
Global	0.09% (0.08–0.11)	0.08% (0.07–0.1)	0.08% (0.07–0.1)	7.44 (6.83–9.13)	7.46 (6.87–9.52)	7.3 (6.74–9.71)
Andean Latin America	0.19% (0.17–0.21)	0.26% (0.23–0.28)	0.38% (0.33–0.39)	0.14 (0.12–0.15)	0.22 (0.19–0.24)	0.34 (0.3–0.35)
Australasia	0.22% (0.19–0.24)	0.2% (0.17–0.21)	0.18% (0.16–0.19)	0.07 (0.06–0.08)	0.07 (0.06–0.08)	0.07 (0.06–0.07)
Caribbean	0.14% (0.09–0.24)	0.18% (0.1–0.36)	0.24% (0.12–0.54)	0.07 (0.04–0.12)	0.09 (0.05–0.18)	0.12 (0.06–0.27)
Central Asia	0.09% (0.07–0.12)	0.08% (0.07–0.12)	0.08% (0.06–0.13)	0.09 (0.07–0.12)	0.09 (0.08–0.14)	0.1 (0.08–0.16)
Central Europe	0.07% (0.07–0.07)	0.05% (0.05–0.05)	0.04% (0.04–0.04)	0.08 (0.07–0.08)	0.05 (0.05–0.05)	0.03 (0.04–0.03)
Central Latin America	0.2% (0.18–0.21)	0.26% (0.23–0.25)	0.33% (0.29–0.3)	0.6 (0.54–0.62)	0.82 (0.73–0.79)	1.08 (0.96–0.98)
Central Sub-Saharan Africa	0.18% (0.07–0.47)	0.28% (0.08–0.91)	0.44% (0.1–1.77)	0.3 (0.11–0.79)	0.58 (0.17–1.91)	1.08 (0.24–4.36)
East Asia	0.21% (0.18–0.23)	0.32% (0.27–0.37)	0.51% (0.42–0.6)	3.09 (2.69–3.51)	4.63 (3.94–5.36)	6.78 (5.58–7.98)
Eastern Europe	0.22% (0.2–0.24)	0.25% (0.23–0.28)	0.29% (0.26–0.31)	0.45 (0.41–0.49)	0.48 (0.44–0.53)	0.52 (0.47–0.58)
Eastern Sub-Saharan Africa	0.17% (0.06–0.49)	0.25% (0.07–0.89)	0.37% (0.08–1.63)	0.93 (0.34–2.72)	1.7 (0.49–6.12)	2.95 (0.67–13.11)
High-income Asia Pacific	0.07% (0.07–0.08)	0.05% (0.05–0.05)	0.03% (0.03–0.04)	0.13 (0.13–0.14)	0.09 (0.08–0.09)	0.05 (0.05–0.06)
High-income North America	0.07% (0.07–0.07)	0.05% (0.05–0.05)	0.03% (0.03–0.03)	0.27 (0.26–0.29)	0.2 (0.19–0.2)	0.14 (0.13–0.14)
North Africa and Middle East	0.16% (0.09–0.34)	0.19% (0.09–0.46)	0.22% (0.1–0.63)	1.2 (0.69–2.5)	1.55 (0.78–3.81)	1.95 (0.86–5.62)
Oceania	0.05% (0.05–0.05)	0.04% (0.05–0.04)	0.04% (0.05–0.04)	0.01 (0.01–0.01)	0.01 (0.01–0.01)	0.01 (0.01–0.01)
South Asia	0.11% (0.09–0.12)	0.14% (0.12–0.16)	0.18% (0.15–0.21)	2.14 (1.88–2.49)	2.86 (2.48–3.39)	3.71 (3.18–4.47)
Southeast Asia	0.08% (0.06–0.1)	0.09% (0.06–0.1)	0.09% (0.07–0.11)	0.58 (0.45–0.72)	0.66 (0.5–0.81)	0.73 (0.53–0.89)
Southern Latin America	0.28% (0.25–0.31)	0.34% (0.3–0.37)	0.42% (0.35–0.46)	0.2 (0.18–0.22)	0.26 (0.22–0.28)	0.32 (0.27–0.35)
Southern Sub-Saharan Africa	0.07% (0.06–0.07)	0.07% (0.07–0.07)	0.08% (0.07–0.08)	0.06 (0.06–0.07)	0.07 (0.07–0.08)	0.08 (0.08–0.09)
Tropical Latin America	0.21% (0.17–0.27)	0.28% (0.22–0.37)	0.36% (0.27–0.51)	0.51 (0.41–0.64)	0.68 (0.53–0.91)	0.88 (0.66–1.25)
Western Europe	0.11% (0.1–0.12)	0.09% (0.08–0.1)	0.07% (0.06–0.07)	0.51 (0.46–0.55)	0.4 (0.36–0.43)	0.31 (0.28–0.32)
Western Sub-Saharan Africa	0.05% (0.04–0.08)	0.05% (0.04–0.09)	0.06% (0.04–0.12)	0.31 (0.24–0.47)	0.44 (0.32–0.75)	0.6 (0.4–1.13)
**(B) Below-Neck Spinal Cord Injuries.**						
	**Age-Standardized Prevalence**			**Cases (Millions)**		
	**2030**	**2040**	**2050**	**2030**	**2040**	**2050**
Global	0.09% (0.07–0.1)	0.08% (0.06–0.09)	0.08% (0.05–0.08)	7.64 (5.95–8.48)	7.52 (5.5–8.3)	7.22 (4.98–7.92)
Andean Latin America	0.2% (0.17–0.26)	0.28% (0.23–0.37)	0.39% (0.31–0.52)	0.15 (0.12–0.19)	0.23 (0.19–0.3)	0.35 (0.27–0.46)
Australasia	0.19% (0.17–0.21)	0.18% (0.16–0.19)	0.16% (0.15–0.17)	0.06 (0.06–0.07)	0.06 (0.06–0.07)	0.06 (0.06–0.06)
Caribbean	0.17% (0.1–0.29)	0.23% (0.11–0.44)	0.3% (0.12–0.66)	0.08 (0.05–0.14)	0.11 (0.06–0.22)	0.15 (0.06–0.33)
Central Asia	0.11% (0.09–0.14)	0.11% (0.08–0.14)	0.11% (0.08–0.15)	0.12 (0.09–0.14)	0.13 (0.09–0.16)	0.14 (0.09–0.18)
Central Europe	0.08% (0.08–0.08)	0.06% (0.06–0.06)	0.04% (0.04–0.04)	0.09 (0.08–0.09)	0.06 (0.06–0.06)	0.04 (0.04–0.04)
Central Latin America	0.23% (0.2–0.26)	0.28% (0.25–0.32)	0.35% (0.31–0.39)	0.67 (0.59–0.76)	0.9 (0.79–1.01)	1.17 (1.03–1.3)
Central Sub-Saharan Africa	0.22% (0.08–0.64)	0.33% (0.1–1.28)	0.52% (0.11–2.53)	0.37 (0.14–1.1)	0.7 (0.2–2.69)	1.28 (0.28–6.25)
East Asia	0.29% (0.25–0.31)	0.48% (0.42–0.52)	0.82% (0.7–0.88)	4.26 (3.78–4.57)	6.96 (6.06–7.42)	11.05 (9.42–11.73)
Eastern Europe	0.33% (0.3–0.37)	0.4% (0.37–0.46)	0.5% (0.45–0.57)	0.66 (0.6–0.74)	0.78 (0.7–0.88)	0.91 (0.82–1.03)
Eastern Sub-Saharan Africa	0.25% (0.08–0.85)	0.39% (0.1–1.68)	0.61% (0.12–3.32)	1.4 (0.45–4.7)	2.69 (0.66–11.52)	4.95 (0.93–26.71)
High-income Asia Pacific	0.06% (0.06–0.07)	0.04% (0.04–0.05)	0.03% (0.03–0.03)	0.11 (0.1–0.12)	0.07 (0.07–0.08)	0.05 (0.04–0.05)
High-income North America	0.06% (0.06–0.07)	0.04% (0.04–0.05)	0.03% (0.03–0.03)	0.24 (0.22–0.26)	0.17 (0.16–0.18)	0.12 (0.11–0.13)
North Africa and Middle East	0.17% (0.1–0.36)	0.19% (0.1–0.46)	0.21% (0.1–0.58)	1.27 (0.74–2.67)	1.57 (0.81–3.79)	1.89 (0.87–5.23)
Oceania	0.05% (0.06–0.05)	0.05% (0.06–0.05)	0.05% (0.06–0.04)	0.01 (0.01–0.01)	0.01 (0.01–0.01)	0.01 (0.01–0.01)
South Asia	0.13% (0.11–0.14)	0.16% (0.14–0.18)	0.2% (0.17–0.22)	2.5 (2.2–2.8)	3.28 (2.86–3.67)	4.16 (3.6–4.67)
Southeast Asia	0.09% (0.07–0.12)	0.09% (0.07–0.13)	0.1% (0.07–0.14)	0.65 (0.49–0.87)	0.73 (0.52–0.98)	0.79 (0.54–1.07)
Southern Latin America	0.27% (0.24–0.28)	0.33% (0.28–0.35)	0.4% (0.34–0.42)	0.19 (0.17–0.21)	0.25 (0.22–0.26)	0.31 (0.27–0.33)
Southern Sub-Saharan Africa	0.09% (0.09–0.1)	0.1% (0.1–0.11)	0.11% (0.12–0.12)	0.09 (0.08–0.09)	0.11 (0.11–0.11)	0.13 (0.13–0.14)
Tropical Latin America	0.25% (0.21–0.32)	0.32% (0.26–0.44)	0.41% (0.33–0.6)	0.58 (0.49–0.76)	0.77 (0.63–1.07)	1 (0.79–1.47)
Western Europe	0.1% (0.09–0.11)	0.07% (0.07–0.08)	0.06% (0.05–0.06)	0.42 (0.4–0.47)	0.33 (0.31–0.36)	0.25 (0.24–0.28)
Western Sub-Saharan Africa	0.07% (0.05–0.11)	0.08% (0.06–0.14)	0.09% (0.06–0.19)	0.42 (0.33–0.69)	0.62 (0.46–1.16)	0.87 (0.61–1.86)

Data in parentheses are 95% uncertainty intervals. Region and super-region numbers do not sum to the global prevalence owing to rounding. YLDs = years lived with disability. Note: Global estimates in the GBD framework are modeled independently and are not the direct sum of regional values. Minor discrepancies may occur owing to separate model calibration, covariate adjustments, and rounding.

## Data Availability

The datasets generated and/or analyzed in the current study are available at https://www.healthdata.org/research-analysis/gbd (accessed on 1 February 2025).

## Supplementary Materials

The following supporting information can be downloaded at: https://www.mdpi.com/article/10.3390/healthcare13202552/s1, Figure S1: Decomposition of changes in years lived with disability (YLDs) attributable to spinal cord injury between 2021 and 2050, stratified by sex and GBD region; Table S1: R Script for Implementation of the Bayesian Multistate Evidence-Synthesis Model; Table S2: Comparison of observed 2021 age-standardized prevalence rates (ASRs) of spinal cord injury with projections derived from models trained on 1990–2020 data; Table S3: Validation of GBD 2021 estimates against registry or published reports in countries with the highest and lowest spinal cord injury (SCI) burden, stratified by lesion level (neck vs. below the neck) and outcome (prevalence, YLDs); Table S4: Correlation of SDI Change with Spinal Cord Injury Prevalence Across GBD Regions, 1990–2021.

## Abbreviations

The following abbreviations are used in this manuscript:

SCI	Spinal cord injury
MS	Multiple sclerosis
GBD	Global burden of disease
YLDs	Years lived with disability
SDI	Sociodemographic Index
HICs	High-income countries
LMICs	Low- and middle-income countries
