# Supplementary material for: Spinal Cord Injury Epidemiology and Causes: A Worldwide Analysis with 2050 Projections
Source: Healthcare (Basel). 2025 Oct 10;13(20):2552. doi: 10.3390/healthcare13202552 (PMC12562789; doi:10.3390/healthcare13202552)

Supplementary Material

Spinal Cord Injury Epidemiology and Causes: A Worldwide Analysis with 2050 Projections

Table S1. R Script for Implementation of the Bayesian Multistate Evidence-Synthesis Model.

```
getwd()
# Install required packages if not already installed:
# install.packages(c("disbayes", "ggplot2", "dplyr"))

library(disbayes)
library(dplyr)
library(ggplot2)

# --------------------------------------------------------------
# 1) Data structure
# --------------------------------------------------------------
# 'disbayes' implements a Bayesian multistate model that links
# incidence, prevalence, remission, and mortality across age
groups.
#
# The dataset must include:
#   - age: integer values, starting at 0 and increasing by 1 year
#   - disease-specific mortality (mandatory)
#   - at least ONE of: incidence or prevalence (mandatory)
#   - remission (optional, only if data are available)
#
# Each measure can be provided in one of three ways:
#   (a) numerator + denominator (e.g., cases, population at risk)
#   (b) point estimate + denominator
#   (c) point estimate + lower + upper bounds (95% CI)
#
# Example skeleton for data frame 'df':
# df <- data.frame(
#   age       = 0:99,
#   inc_num    = ..., inc_denom = ...,
#   prev_num   = ..., prev_denom = ...,
#   mort_num   = ..., mort_denom = ...,
#   # Optional remission:
#   # rem_num = ..., rem_denom = ...
# )

# --------------------------------------------------------------
# 2) Converting prevalence with CI into counts
# --------------------------------------------------------------
# If only prevalence and a 95% confidence interval are available
# (without raw counts), the function ci2num() can reconstruct
# an approximate numerator and denominator consistent with
# a Beta distribution posterior.
#
# Example: prevalence = 0.12 with 95% CI: 0.10-0.14
conv <- ci2num(est = 0.12, lower = 0.10, upper = 0.14)
# conv$num and conv$denom can then be inserted into the dataset.

# --------------------------------------------------------------
# 3) Fit the Bayesian multistate evidence-synthesis model
```

```
# --------------------------------------------------------------
# Key settings:
#   - inc_model, cf_model: choose "smooth" for age-spline smoothing
#   - rem_model: choose "const" to assume constant remission across
ages
#   - sprior: hyperprior for spline smoothness penalty
#   - eqage: sets an age range to be treated as equal (stabilizes
estimates)
#   - method: "mcmc" for full Bayesian inference via Stan,
#            "opt" for optimization-based approximation
#
# Replace column names below with the actual names from your
dataset.
fit <- disbayes(
  data      = df,
  inc_num   = "inc_num",    inc_denom = "inc_denom",
  prev_num  = "prev_num",   prev_denom = "prev_denom",
  mort_num  = "mort_num",   mort_denom = "mort_denom",
  age      = "age",
  inc_model = "smooth",
  cf_model  = "smooth",
  rem_model = "const",
  sprior    = c(inc = 1, cf = 1, rem = 1),
  eqage     = 30,
  method    = "mcmc",
  iter      = 4000,
  stan_control = list(adapt_delta = 0.95, max_treedepth = 12)
)

# --------------------------------------------------------------
# 4) Summarize and visualize results
# --------------------------------------------------------------
# Extract posterior summaries for all parameters (by age):
post <- tidy(fit)
# Example parameters include:
#   - inc_prob: incidence probability per year
#   - prev_prob: prevalence probability
#   - mort_prob: disease-specific mortality probability
#   - cf_prob: case fatality probability

# Quick plot of prevalence by age with 95% credible intervals:
plot(fit, variable = "prev") + theme_minimal()

# Compare observed data against model predictions (model fit
diagnostic):
plotfit_disbayes(fit, agemin = 0)
```

Table S2. Comparison of observed 2021 age-standardized prevalence rates (ASRs) of spinal cord injury with projections derived from models trained on 1990–2020 data. Values are presented by GBD region and lesion level (cervical vs. below cervical), including observed ASRs, predicted ASRs, 95% prediction intervals (PIs), absolute differences, and relative errors (%).

(A) Neck-level spinal cord injuries.

Region	Observed 2021 ASR (%)	Predicted 2021 ASR (%)	95% Prediction Interval	Absolute Difference	Relative Error (%)
**Oceania**	25.820	25.840	(22.96-28.41)	0.020	0.100
**Southern Sub-Saharan Africa**	24.940	24.750	(22.55-26.86)	0.190	0.800
**Andean Latin America**	37	36.600	(35.71-37.4)	0.410	1.100
**Central Asia**	43.710	43.180	(40.12-46.31)	0.530	1.200
**High-income North America**	95.510	94.270	(89.95-98.99)	1.240	1.300
**North Africa and Middle East**	60.380	61.200	(59.12-63.29)	0.830	1.400
**Central Europe, Eastern Europe, and Central Asia**	78.900	77.790	(71.04-85.42)	1.100	1.400
**East Asia**	47.210	46.480	(41.54-51.67)	0.720	1.500
**Southeast Asia, East Asia, and Oceania**	42.520	41.860	(38.48-45.26)	0.660	1.500
**Southeast Asia**	32.920	32.380	(29.91-35.0)	0.540	1.700
**Tropical Latin America**	58.800	57.580	(55.37-59.6)	1.220	2.100
**Eastern Europe**	90	87.740	(75.75-98.77)	2.270	2.500
**Central Europe**	88.890	86.640	(83.15-90.66)	2.250	2.500
**Australasia**	139.680	136.030	(132.23-139.91)	3.650	2.600
**Global**	48.930	47.600	(46.19-49.25)	1.330	2.700
**Southern Latin America**	94.050	91.490	(87.89-95.02)	2.560	2.700
**High-income**	104.490	101.600	(98.72-104.74)	2.890	2.800
**Western Sub-Saharan Africa**	17.290	16.770	(16.04-17.52)	0.520	3
**Latin America and Caribbean**	52.560	50.860	(47.04-55.09)	1.700	3.200
**South Asia**	29.580	28.620	(27.57-29.74)	0.970	3.300
**Western Europe**	113.750	110.030	(107.21-112.82)	3.720	3.300
**Central Latin America**	51.530	49.570	(40.52-60.55)	1.960	3.800
**High-income Asia Pacific**	98.530	94.230	(89.87-98.21)	4.310	4.400
**Sub-Saharan Africa**	20.670	19.420	(16.79-22.01)	1.250	6
**Central Sub-Saharan Africa**	23.060	21.500	(16.8-26.44)	1.560	6.800
**Eastern Sub-Saharan Africa**	22.980	21.110	(14.24-28.39)	1.870	8.100
**Caribbean**	50.240	55.500	(35.37-74.53)	5.260	10.500

(B) Below-neck spinal cord injuries.

Region	Observed 2021 ASR (%)	Predicted 2021 ASR (%)	95% Prediction Interval	Absolute Difference	Relative Error (%)
Oceania	29.760	29.780	(26.83-32.91)	0.020	0.100
North Africa and Middle East	69.270	70.030	(67.78-72.31)	0.760	1.100
Andean Latin America	41.710	41.200	(40.2-42.03)	0.510	1.200
Southeast Asia	38.340	37.820	(35.34-40.59)	0.520	1.300
Central Asia	50.940	50.150	(46.54-54.16)	0.790	1.600
High-income North America	85.750	84.080	(79.25-88.69)	1.670	1.900
Central Europe, Eastern Europe, and Central Asia	92.720	90.780	(83.66-97.92)	1.940	2.100
Southeast Asia, East Asia, and Oceania	47.320	46.260	(41.96-50.53)	1.070	2.300
Southern Sub-Saharan Africa	29.780	29.080	(27.26-30.68)	0.700	2.300
Australasia	119.760	116.850	(113.46-120.25)	2.920	2.400
East Asia	51.750	50.400	(43.69-57.02)	1.350	2.600
Tropical Latin America	65.590	63.870	(61.32-66.59)	1.730	2.600
Central Europe	102.480	99.710	(95.72-103.82)	2.780	2.700
Western Sub-Saharan Africa	20.160	19.570	(18.78-20.45)	0.590	2.900
Latin America and Caribbean	58.930	57.160	(52.26-62.14)	1.760	3
Southern Latin America	84.940	82.370	(79.77-85.17)	2.570	3
High-income	90.880	88.080	(84.91-91.17)	2.800	3.100
Western Europe	97.210	93.970	(90.94-96.98)	3.240	3.300
Eastern Europe	107.060	103.330	(93.07-113.92)	3.730	3.500
Global	52.200	50.380	(48.4-52.5)	1.820	3.500
South Asia	34.870	33.610	(32.48-34.79)	1.260	3.600
Central Latin America	57.750	55.390	(44.77-68.03)	2.360	4.100
High-income Asia Pacific	83.580	79.950	(75.95-84.01)	3.640	4.400
Sub-Saharan Africa	25.190	23.950	(21.15-26.75)	1.240	4.900
Central Sub-Saharan Africa	28.480	27.020	(21.82-32.37)	1.460	5.100
Eastern Sub-Saharan Africa	29.020	27.210	(20.27-34.85)	1.810	6.200
Caribbean	57.680	63.510	(42.36-85.31)	5.830	10.100

Table S3. Validation of GBD 2021 estimates against registry or published reports in countries with the highest and lowest spinal cord injury (SCI) burden, stratified by lesion level (neck vs. below the neck) and outcome (prevalence, YLDs).

Measure	Rank	location_name	value_label
Prevalence - Neck level	High	Syrian Arab Republic	385.72249 (165.95347 - 805.98997)
Prevalence - Neck level	High	Islamic Republic of Afghanistan	326.10404 (139.05754 - 702.11688)
Prevalence - Neck level	Low	Republic of Madagascar	26.32919 (24.33234 - 28.70243)
Prevalence - Neck level	Low	Republic of Malawi	25.39672 (23.55869 - 27.82718)
YLDs - Neck level	High	Syrian Arab Republic	153.01957 (63.79718 - 349.05985)
YLDs - Neck level	High	Islamic Republic of Afghanistan	140.49211 (61.3723 - 299.21487)
YLDs - Neck level	Low	Republic of Madagascar	11.70704 (8.26103 - 15.01864)
YLDs - Neck level	Low	Republic of Malawi	11.25456 (8.27272 - 14.4232)
Prevalence - Below neck	High	Islamic Republic of Afghanistan	461.11717 (174.38841 - 1126.6574)
Prevalence - Below neck	High	Syrian Arab Republic	430.32813 (181.94918 - 934.68257)
Prevalence - Below neck	Low	Republic of Madagascar	32.21588 (29.35197 - 35.22208)
Prevalence - Below neck	Low	Republic of Malawi	32.02518 (29.60312 - 34.78056)
YLDs - Below neck	High	Islamic Republic of Afghanistan	136.04089 (53.37208 - 334.29193)
YLDs - Below neck	High	Syrian Arab Republic	88.97633 (37.12823 - 211.83822)
YLDs - Below neck	Low	Kingdom of Tonga	9.25019 (6.4235 - 12.2831)
YLDs - Below neck	Low	Taiwan (Province of China)	8.50125 (5.71864 - 11.73481)

Table S4. Correlation of SDI Change with Spinal Cord Injury Prevalence Across GBD Regions, 1990–2021

GBD Region	n	SDI Range	SDI Change (%)	SCI Prevalence Range	SCI Change (%)	R^2	Correlation (r)	p-value
**Oceania**	32	0.391-0.467	19.5	72.65-106.15	46.1	0.932***	0.965	<0.001
**Southern Sub-Saharan Africa**	32	0.507-0.642	26.7	101.82-140.36	-26.7	0.838***	-0.915	<0.001
**South Asia**	32	0.320-0.558	74.4	99.05-123.61	24.8	0.829***	0.91	<0.001
**Caribbean**	32	0.518-0.642	23.9	120.85-212.39	72.3	0.806***	0.898	<0.001
**Western Europe**	32	0.746-0.849	13.7	404.81-467.75	-12.7	0.770***	-0.877	<0.001
**High-income Asia Pacific**	32	0.768-0.877	14.2	347.82-424.43	-14.9	0.767***	-0.876	<0.001
**North Africa and Middle East**	32	0.437-0.658	50.5	214.18-253.55	17.1	0.750***	0.866	<0.001
Southeast Asia	32	0.464-0.650	40	124.89-140.48	10.2	0.727***	0.852	<0.001
**Central Asia**	32	0.553-0.675	22	180.17-197.95	-5.6	0.682***	-0.826	<0.001
**Southern Latin America**	32	0.587-0.736	25.3	292.45-355.44	17.7	0.635***	0.797	<0.001
**Eastern Sub-Saharan Africa**	32	0.234-0.410	75.4	99.40-140.55	-14.3	0.623***	-0.789	<0.001
**High-income North America**	32	0.766-0.863	12.8	329.64-394.45	-11.8	0.540***	-0.735	<0.001
**Eastern Europe**	32	0.664-0.803	20.9	368.30-444.70	-9.7	0.447***	-0.669	<0.001
**East Asia**	32	0.471-0.726	54	140.42-192.06	33.8	0.444***	0.666	<0.001
**Western Sub-Saharan Africa**	32	0.274-0.446	63	63.01-70.59	12	0.409***	0.639	<0.001
**Andean Latin America**	32	0.500-0.652	30.3	139.22-156.65	9.5	0.381***	0.617	<0.001
**Australasia**	32	0.731-0.846	15.6	500.05-556.25	-7.2	0.368***	-0.607	<0.001
**Tropical Latin America**	32	0.500-0.652	30.6	210.29-245.59	12.9	0.306**	0.554	0.001
**Central Sub-Saharan Africa**	32	0.302-0.472	56.2	87.08-129.19	12.2	0.026	-0.162	0.375
**Central Latin America**	32	0.486-0.641	31.9	206.96-248.94	-11.6	0.009	0.093	0.613
**Central Europe**	32	0.637-0.796	24.9	364.01-398.24	0.9	0.008	0.09	0.622

Figure S1. Decomposition of changes in years lived with disability (YLDs) attributable to spinal cord injury between 2021 and 2050, stratified by sex and GBD region. Bars represent the contributions of population aging (blue), population growth (green), and changes in age-specific YLD rates (yellow) to the total percentage change, with black dots denoting the net change. Note: X-axis values represent percentage changes multiplied by 100 for visualization; actual relative changes should be interpreted accordingly.

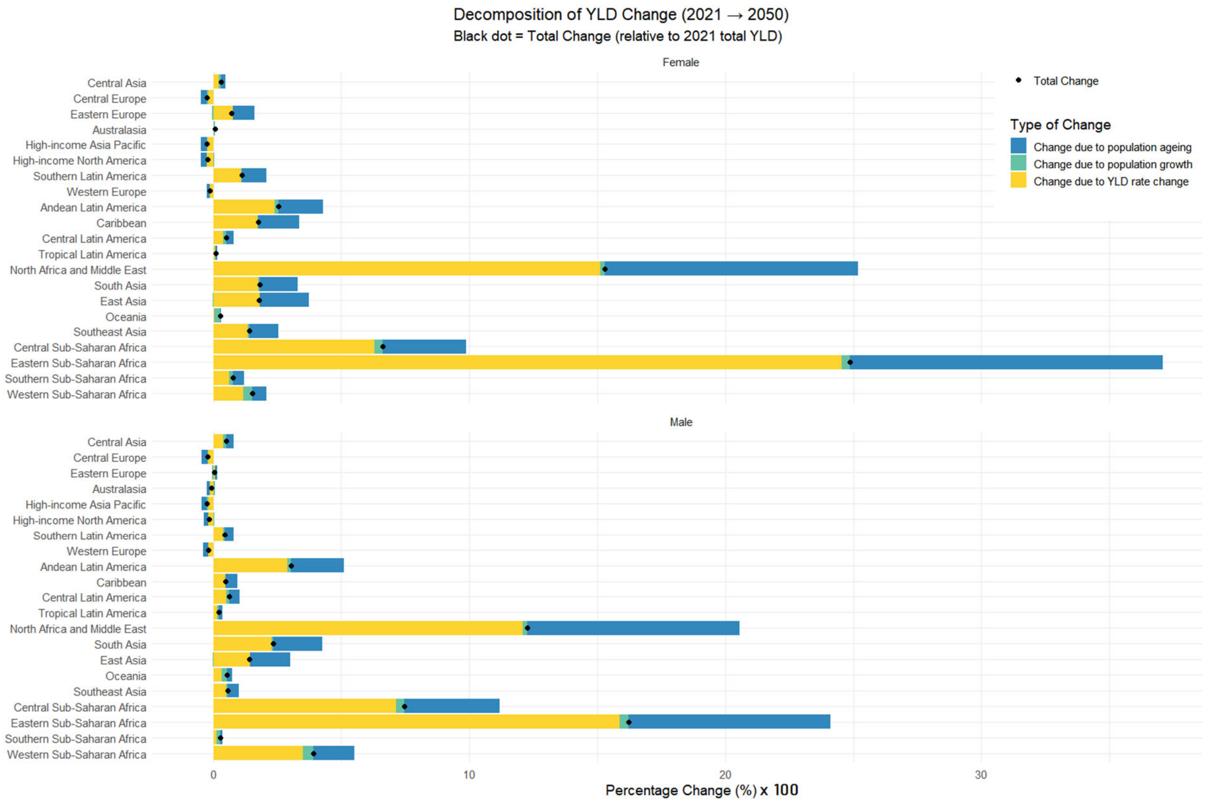

Supplement: Supplementary file 1 [file healthcare-13-02552-s001.zip › healthcare-3872731-supplementary.pdf]
